# Semiautomatic Treatment Planning for the Field-in-field Technique in Whole Brain Irradiation

**DOI:** 10.14789/jmj.JMJ22-0003-OA

**Published:** 2022-08-02

**Authors:** HIROYUKI WATANABE, SATORU SUGIMOTO, TORU KAWABATA, HIRONORI NAGATA, CHIE KUROKAWA, KEISUKE USUI, TATSUYA INOUE, JUN TAKATSU, KYOICHI KATO, KEISUKE SASAI

**Affiliations:** 1 Department of Radiation Oncology, Graduate School of Medicine, Juntendo University, Tokyo, Japan; 1 Department of Radiation Oncology, Graduate School of Medicine, Juntendo University, Tokyo, Japan; 2 Graduate School of Health Sciences, Showa University, Kanagawa, Japan; 2 Graduate School of Health Sciences, Showa University, Kanagawa, Japan; 3 Department of Radiation Oncology, Faculty of Medicine, Juntendo University, Tokyo, Japan; 3 Department of Radiation Oncology, Faculty of Medicine, Juntendo University, Tokyo, Japan; 4 Department of Radiation Oncology, Shonan Kamakura General Hospital, Kanagawa, Japan; 4 Department of Radiation Oncology, Shonan Kamakura General Hospital, Kanagawa, Japan; 5 Department of Radiological Technology, Faculty of Health Science, Juntendo University; 5 Department of Radiological Technology, Faculty of Health Science, Juntendo University

**Keywords:** automation, radiotherapy treatment planning, whole brain irradiation

## Abstract

**Objectives:**

In radiation therapy, the field-in-field (FIF) technique is used to prevent the administration of unnecessarily high doses to reduce toxicity. Recently, the FIF technique has been used for whole brain irradiation (WBI). Using the FIF technique, the volume that receives a higher than prescribed dose (hotspot) can be largely reduced; however, the treatment planning requires time. Therefore, to reduce the burden on the treatment planners, we propose a semiautomatic treatment planning method for the FIF technique.

**Methods:**

In the semiautomatic FIF technique, hotspot regions in a treatment plan without the FIF technique are identified three-dimensionally, and beams with blocks that cover the hotspot regions using a multileaf collimator (sub-beams) are automatically created. The sub-beams are added to the original plan, and weights are assigned based on the maximum dose of the original plan to decrease the doses in the hotspot regions. This method was applied to 22 patients previously treated with WBI, wherein treatment plans were originally created without the FIF technique.

**Results:**

In the semiautomatic FIF plans, the hotspots almost disappeared. The dose to 95% of the volume and the volume receiving at least 95% of the prescribed dose in the planning target volume decreased by only 0.3% ± 0.2% and 0.0% ± 0.1%, respectively, on average compared with those in the original plan. The average semiautomatic FIF processing time was 28 ± 4 s.

**Conclusions:**

The proposed method reduced the hotspot regions with a slight change in the target coverage.

## Introduction

Whole brain irradiation (WBI) is one of the main treatments for patients with brain metastases and is also performed for prophylactic cranial irradiation as an adjuvant therapy for patients with small cell lung cancers^1-3)^. WBI is usually performed using opposed lateral fields. Treatment with opposed lateral fields generates a hotspot in the frontal lobe, where the dose is considerably higher than the prescribed dose^2, 4)^. Recently, the uniformity of the target dose has been improved by introducing advanced techniques, such as intensity-modulated radiation therapy. The International Commission on Radiation Units and Measurements (ICRU) recommends that the dose in the planning target volume (PTV) should be 95%-107% of the prescribed dose^5)^. In this situation, a method to reduce hotspots should be used, even in WBI, to reduce the risk of cognitive impairment as much as possible.

The field-in-field (FIF) technique has been used to reduce unnecessarily high doses^6, 7)^. To apply the FIF technique to WBI, several beams are added to the opposed lateral fields as sub-beams. In the sub-beams, the hotspot regions are blocked to reduce the dose in these regions^7-9)^. In this technique, additional time is required to create the sub- beams manually. If treatment planning using the FIF technique is automated, the burden of treatment planners is greatly reduced without compromising the quality of the treatment plans^10, 11)^.

Several studies have been conducted on automatic planning for conventional techniques, including the FIF technique^11-14)^. Kim et al. retrieved Digital Imaging and Communications in Medicine (DICOM) data from a treatment planning system (TPS) and created FIF plans for breast-conserving therapy^12)^. Yu et al. developed an automatic multileaf collimator shaping technique for WBI using deep learning^14)^. They adopted a simple two-opposing-lateral-field technique, and therefore, obtained the relatively high average max dose of approximately 110%.

In this study, we propose a semiautomatic treatment planning method for the FIF technique for WBI using an application programming interface provided by a TPS manufacturer without outputting DICOM data outside the TPS.

## Materials and Methods

### Overview of the proposed method

**Figure 1:**
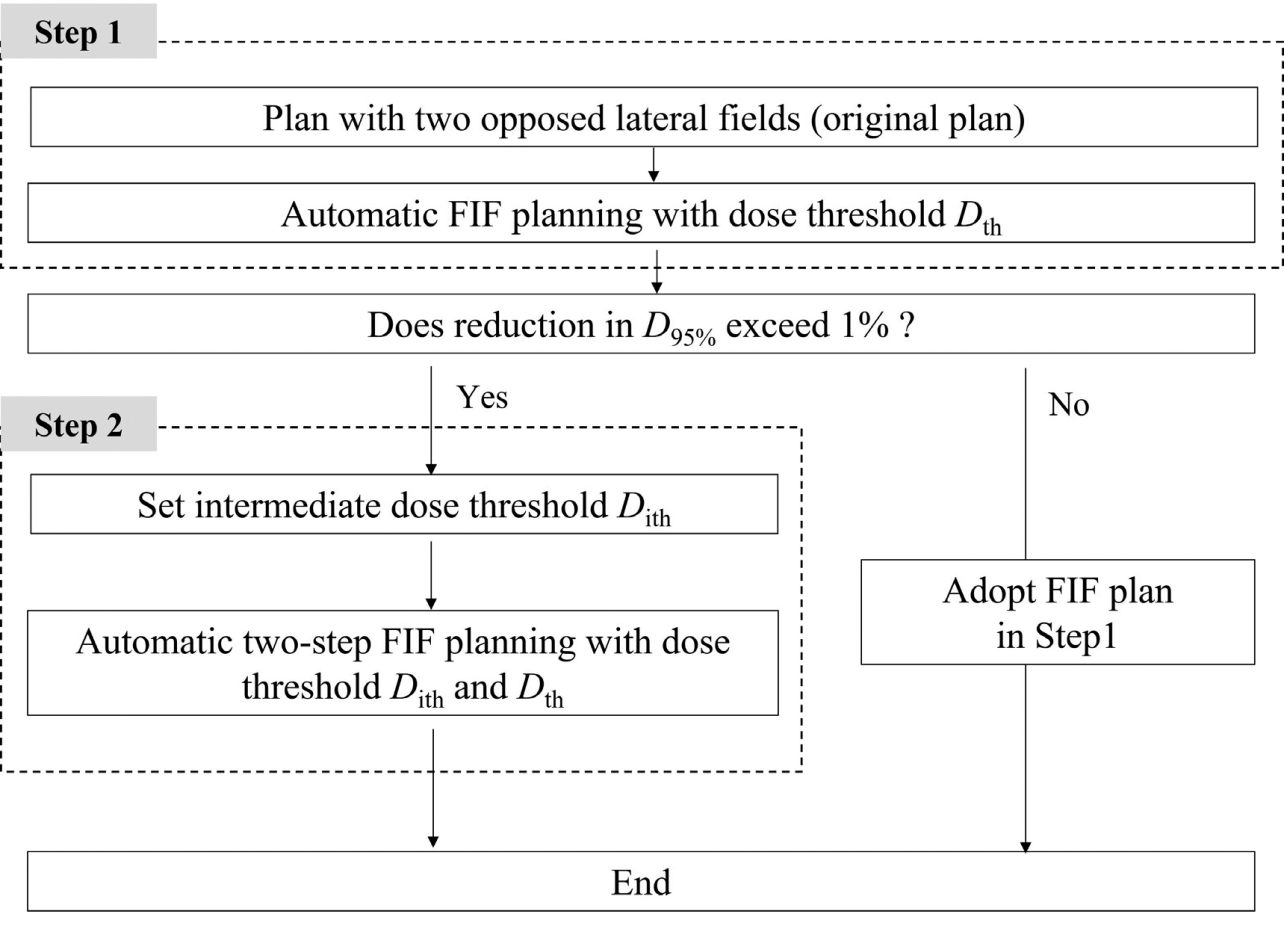
Flowchart of the semiautomatic field-in-field (FIF) technique *D*_th_ is the dose threshold for the hotspot regions and fixed at 105% of the prescribed dose at the isocenter in this study. *D*_95%_ is the dose covering 95% of the PTV. *D*_max_ is the maximum dose in the original plan. *D*_ith_ is the intermediate threshold defined as *D*_ith_=(*D*_max_+*D*_th_)/2.

In the FIF technique for WBI, we developed an automatic technique for creating sub-beams, and adjusting beam weights to make hotspot regions disappear. Because we only automated the sub-beams creation and weight adjusting for the FIF technique, we describe our method as semiautomatic. In an automatic planning, whole planning process from the creation of main beams will be automated. The hotspot regions were defined as regions receiving dose above a predefined threshold *D*_th_. Figure 1 shows an outline of the proposed method. The input of the method (an original treatment plan) was a conventional treatment plan using two-opposing-lateral fields for WBI, which was created in a TPS (Eclipse version 11.0; Varian Medical Systems, USA). The method consists of two steps: in Step 1, an algorithm for creating sub-beams and adjusting beam weights is applied to the original treatment plan and an FIF plan with two sub-beams is created with a predefined threshold *D*_th_ for hotspot regions; and in Step 2, the dose index of the PTV in the FIF plan is evaluated to determine whether further adjustment is required. When the reduction in the *D*_95%_ which represents the dose covering 95% of the PTV in Step 1 exceeds 1%., FIF treatment planning with four sub-beams is performed to alleviate the reduction of the *D*_95%_.

Steps 1 and 2 were implemented in a research version of the TPS (Eclipse, version 13.7; Varian Medical Systems, USA) using the Eclipse Scripting Application Programming Interface (ESAPI) and automatically performed.

The programming language used in the ESAPI was C# (Microsoft Corporation, USA). The research version of Eclipse (13.7) was used because Eclipse version 11.0 did not allow a change of a treatment plan using ESAPI. We retrospectively applied the automatic FIF script to the treatment plans of patients treated using the two-opposing-lateral-field technique.

### Automatic FIF technique

#### FIF plan in Step 1

**Figure 2:**
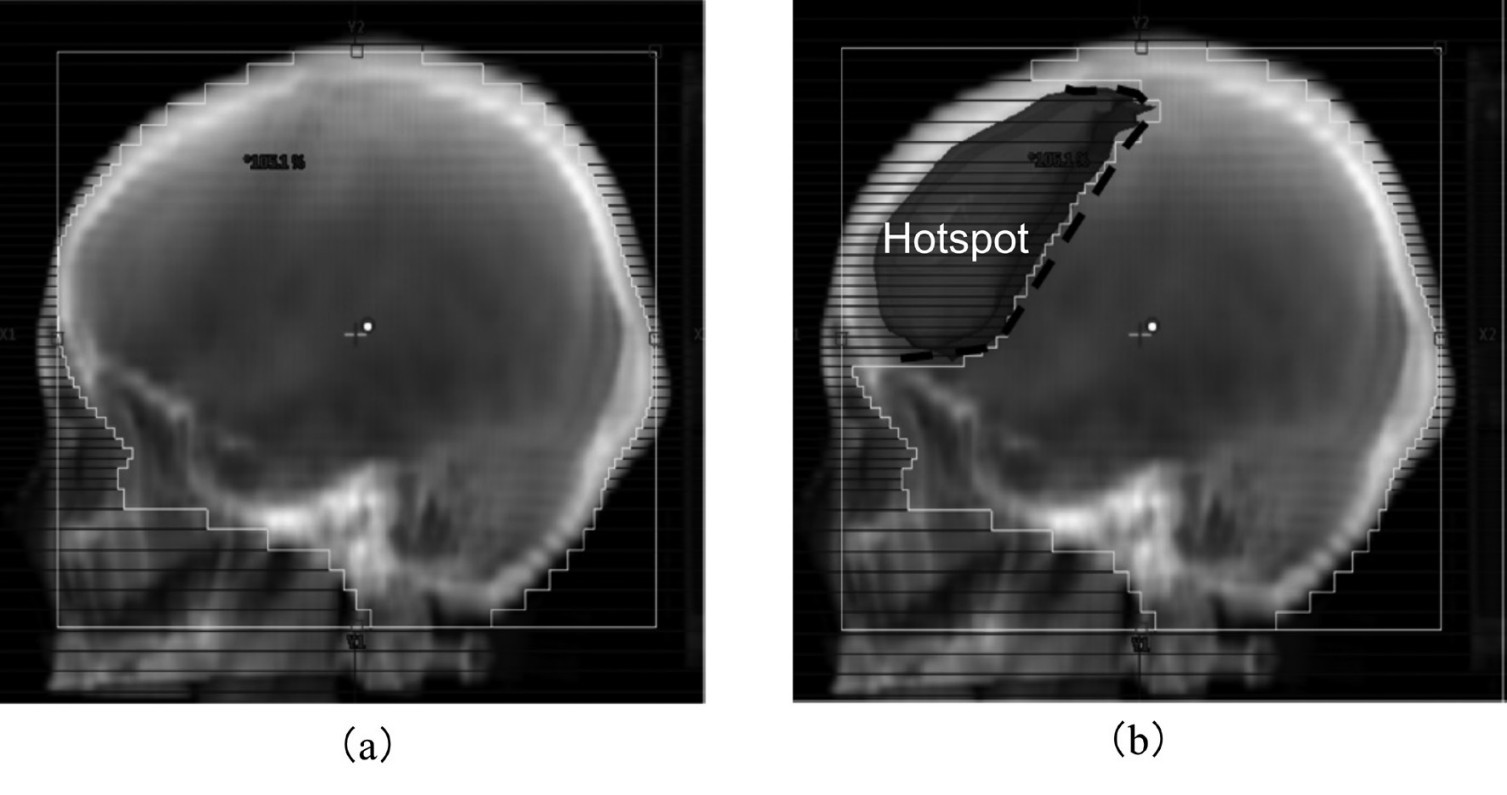
Beam's eye views with the multileaf collimator shapes of (a) the main beam and (b) sub-beam in the field-in-field plan. The projection of the hotspot region in the original plan is indicated by Hotspot in (b).

In the original treatment plan, hotspot regions that received a dose above *D*_th_ were identified in the three-dimensional (3D) dose distribution calculated in the TPS. The two beams in the original plan (main beams) were duplicated as sub-beams, which initially had the same multileaf collimator (MLC) positions as the main beams and no beam weights. In the FIF technique, the fields of sub-beams are shaped to block the hotspot region using the MLC. Figure 2 shows the beam’s eye views (BEVs) of (a) one of the main beams and (b) the corresponding sub-beam. The positions of the MLC in the sub-beams were determined to block the hotspot region without a margin in the BEV. Because the hotspot region tends to be located in the frontal and occipital lobes at the edge of the irradiation field, the isocenter was not blocked in the sub-beams in almost all cases.

**Figure 3:**
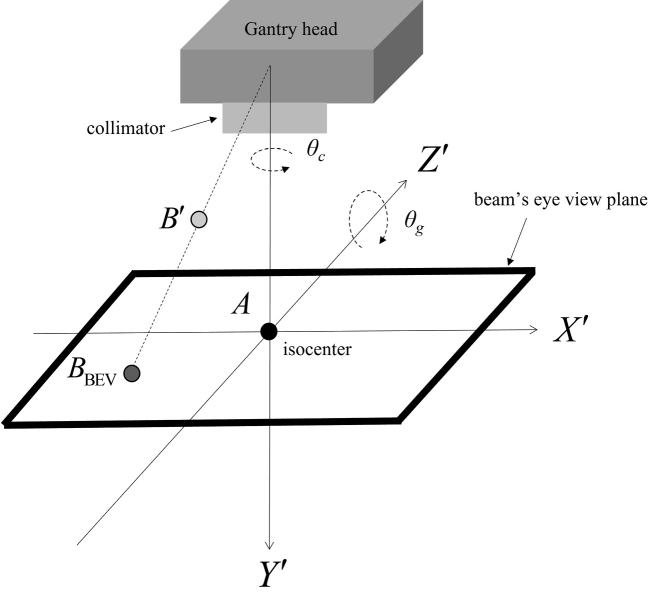
Definition of the beam coordinate system (BCS) and beam's eye view (BEV) plane, which are fixed to the collimator and rotate with the collimator and gantry. When both the collimator and gantry angles are 0°, the *X'* axis is in the cross-plane direction, *Y'* axis is in the vertical direction, *Z'* axis is in the in-plane direction, rotation of the collimator is around the *Y'* axis (θ_c_), and rotation of the gantry is around the *Z'* axis (θ_g_). Point *A* is the isocenter. By projecting onto the BEV plane, the point *B'* (*X'*, *Y'*, *Z'*) is moved to *B*_BEV_ (*X*_BEV_, 0, *Z*_BEV_).

**Figure 4:**
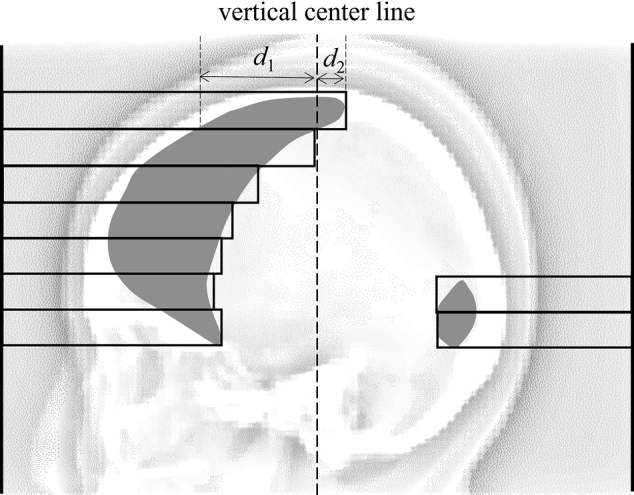
Schematic beam's eye view of a sub-beam. The gray regions indicate projected hotspots The dotted line is the vertical center line of the field of the sub-beam. Rectangles indicate multileaf collimator leaves, which completely cover the hotspots. *d*_1_ (*d*_2_) is the horizontal distance from the vertical center line to the outer edges of the hotspot on the left (right) side.

To identify the hotspot region, the 3D dose distribution of the original plan was extracted using the ESAPI. In the ESAPI, the 3D dose distribution was defined in the DICOM coordinate system (DCS) fixed in a patient. The hotspot region defined in the DCS was projected onto the BEV using the following three steps: (1) the coordinate transformation of translation from the DCS (*x*, *y*, *z*) to the coordinate system (*X*, *Y*, *Z*), where the isocenter was at the origin (isocenter coordinate system, ICS), was performed.



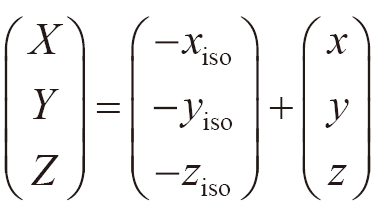
,


where (*x*_iso_, *y*_iso_, *z*_iso_) are the coordinates of the isocenter in the DCS. (2) The coordinate transformation from the ICS to a coordinate system (*X′*, *Y′*, *Z′*) fixed at the gantry with the isocenter as the origin (beam coordinate system [BCS]) was performed.



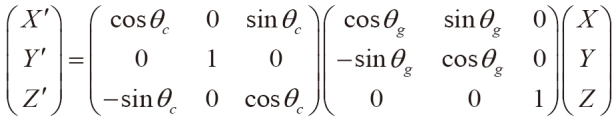
,


where θ_c_ and θ_g_ are the collimator and gantry angles, respectively. The senses of rotation of the collimator and gantry followed the IEC61217 scale convention, and the directions of the *X′*, *Y′*, and *Z′* axes are presented in Figure 3. (3) A point in the BCS was projected onto the BEV plane at the isocenter (*Y′* = 0 in the BCS) as follows:



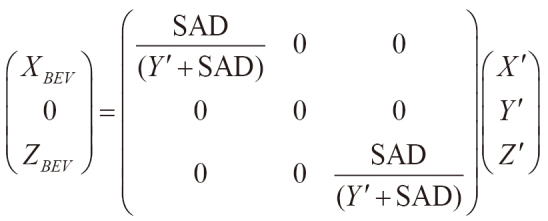
,


where SAD is the source-to-isocenter distance of 100 cm. With the projection, point *B′* was moved to point *B*_BEV_, as shown in Figure 3. By projecting all hotspots in the 3D dose distribution, the projection of the hotspot region on the BEV was obtained as a binary image (Figure 4).

The MLC leaves in the sub-beams were moved to cover the hotspot region in the BEV as in Figure 4. An MLC leave which is on the side of a hotspot was used to block the hotspot if the hotspot did not cross the vertical center line of a field. If a hotspot region crossed the vertical center line of a field, the horizontal distances from the center of the field to the outer edges of the hotspot were measured, as *d*_1_ and *d*_2_ in Figure 4. The side of an MLC leave which had a longer horizontal distance (*d*_1_ in Figure 4) than the other side was selected to block the hotspot. The MLC leaves were moved as far as completely covering the hotspot.

The weights of the main beams were reduced to decrease the dose in the hotspot regions as follows:



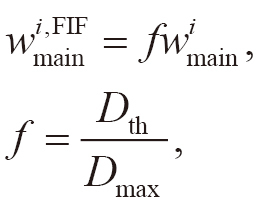
　　(1)


where *D*_max_ is the maximum dose in the original plan, *D*_th_ is the dose threshold for the hotspot regions, *i* is the index for the main beams (*i* =1, 2), and $w_{\mathrm{main}}^{i}$ and $w_{\mathrm{main}}^{\mathrm{i,FIF}}$ are the weights in the original and FIF plans for the main beams, respectively. The weights of the sub-beams ($w_{\mathrm{sub}}^{\mathrm{i,FIF}}$, *i* =1, 2) were assigned as follows:



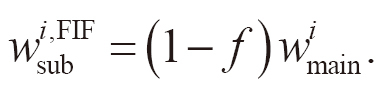
　　(2)


Approximately, with the new weights, $w_{\mathrm{main}}^{i}$ and $w_{\mathrm{sub}}^{\mathrm{i,FIF}}$, the doses in the unblocked regions do not change and those in the blocked regions reduced by *f*.

With the new MLC shapes for the sub-beams and new weights for the main beams and sub-beams, the 3D dose distribution for the FIF plan with the two sub-beams (FIF) was calculated in the TPS and dose indices were evaluated. In this study, *D*_th_ was fixed at 105% of the prescribed dose at the isocenter.

#### FIF plan in Step 2

After Step 1, when the dose coverage of the target is greatly reduced, there is a possibility that the reduction in the dose coverage can be alleviated by increasing the number of sub-beams. This is because an increase in the number of sub-beams provides higher degrees of freedom for dose modification. In this study, when the reduction in *D*_95%_ of the PTV was >1% in Step 1, FIF treatment planning with four sub-beams was performed. The criterion of 1% reduction was arbitrarily adopted as an example. It can be changed to a different criterion easily.

**Figure 5:**
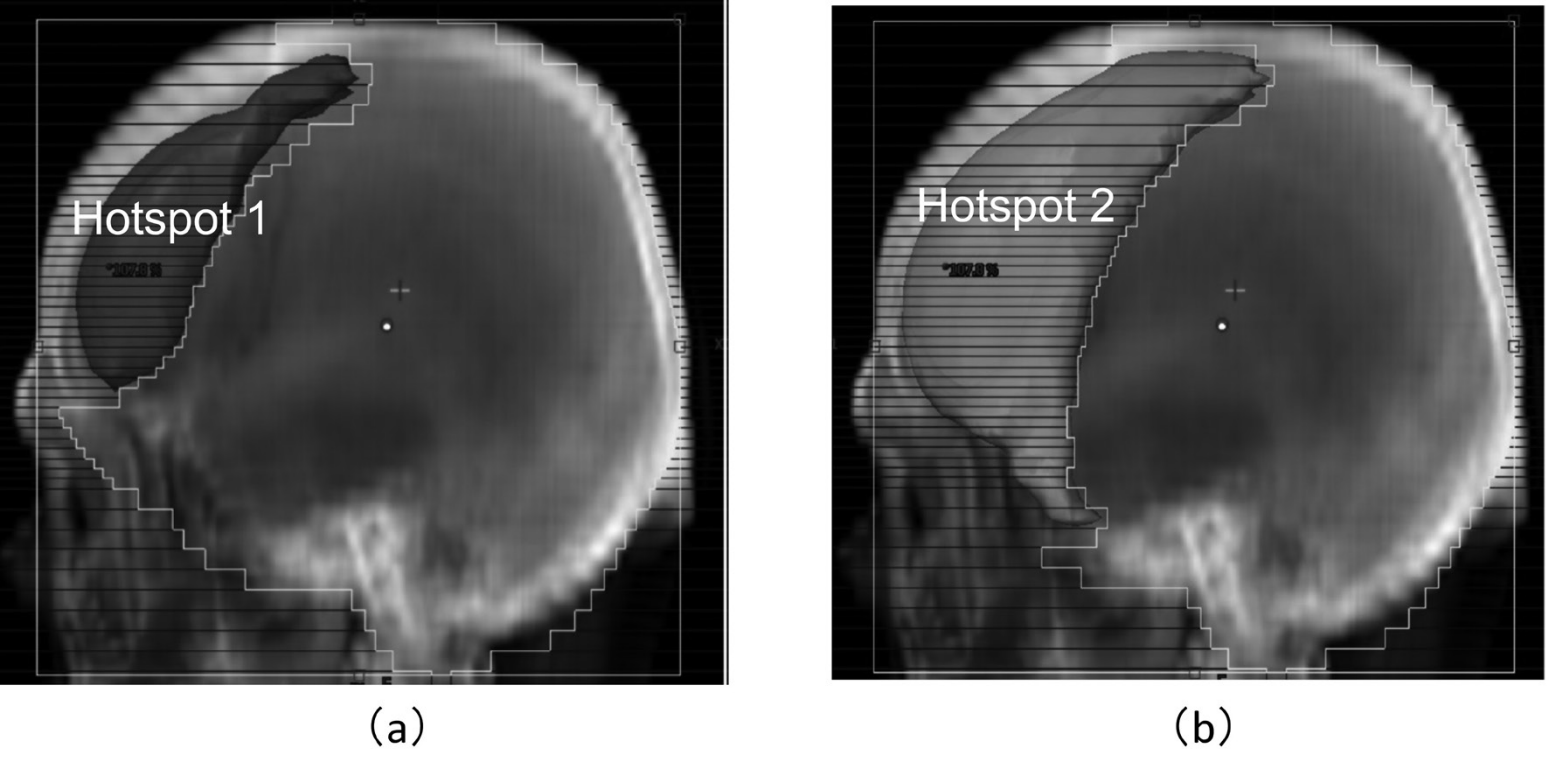
Beam's eye views (BEVs) with the multileaf collimator shapes of the sub-beams in the four-sub-beam field-in-field plan. (a) The BEV of one of the first sub-beams. The high-hotspot region of 106.4 % of the prescribed dose in the original plan is represented by Hotspot 1. (b) The BEV of one of the second sub-beams. The low-hotspot region of 105 % of the prescribed dose after the first step is indicated as Hotspot 2.

In this step, the sub-beams were added sequentially. First, two sub-beams were added to the original plan, as in Step 1. The high-hotspot regions that received a dose greater than the intermediate threshold *D*_ith_=(*D*_max_+*D*_th_)/2 were identified in the dose distribution in the original plan, as shown in Figure 5a. The same procedures as in Step 1 were performed by replacing *D*_th_ with *D*_ith_. After the procedures, the FIF plan with the two sub-beams was obtained. Second, two sub-beams were newly added. The low-hotspot regions that received a higher dose than *D*_th_ were identified in the FIF plan with the two sub-beams. The MLC apertures of the two extra sub-beams were shaped to block the low-hotspot regions in the BEV, as shown in Figure 5b.

The weights for the main beams and the first two sub-beams were assigned, as shown in Eqs. (1) and (2), where *D*_th_ is replaced by *D*_ith_. The weights for the main beams $w_{\mathrm{main}}^{\mathrm{i,FIF2nd}}$ and extra sub-beams $w_{\mathrm{sub}}^{\mathrm{i,FIF2nd}}$ in the four sub-beams FIF plan were assigned as follows:




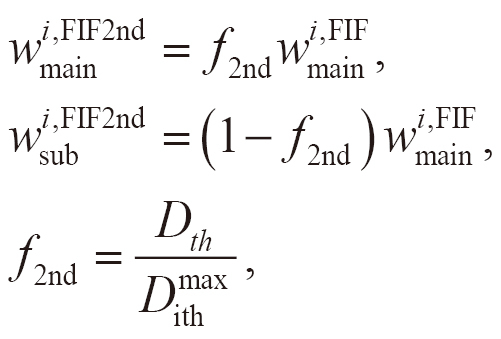




where *i* is the index for the main beams (*i* =1, 2), and $w_{\mathrm{ith}}^{\max}$ is the maximum dose in the FIF plan with the two sub-beams. The 3D dose distribution was calculated for the FIF plan with four sub-beams (FIF-4SF), and the dose indices were evaluated.

### Application of the automated FIF algorithm

#### Patients

We used the planning data of 22 patients with brain metastases from lung, breast, and rectal cancers treated with WBI from January 2016 to December 2017 at Showa University Yokohama Northern Hospital. The study protocol was approved by the institutional review board (##17HO92) of our hospital, and the need for informed consent was waived. This study was conducted in accordance with the Declaration of Helsinki and Japanese ethical guidelines for epidemiologic research.

The clinical treatment plans were used as the original plan for the automated FIF algorithm. All plans used two laterally opposing fields with 10 MV of X-ray energy and prescribed a total dose of 30 Gy (2 Gy per fraction) or 31.2 Gy (1.2 Gy per fraction delivered twice daily) at the isocenter. The dose calculation algorithm used for all plans was an analytical anisotropic algorithm^15)^ in the TPS (Eclipse, version 13.7.14; Varian Medical Systems, USA). The PTV included the cerebral parenchyma, with a margin of 2 or 3 mm. The original plans were created for a linac (TrueBeam STx; Varian Medical Systems, USA) with the MLC containing 14 pairs of leaves with a 2.5-mm width and 32 pairs of leaves with a 5-mm width. The dose grid size was 2.5×2.5×2.5 mm^3^ in the TPS.

#### Creation of a manual FIF plan for comparison with automatic FIF

We also manually created FIF plans from the original plans by a board-certified physicist . Two sub-beams were created by copying the two main beams. Hotspot regions in the original plan were blocked using MLC leaves in both sides of the sub-beams. The hotspot regions were visually identified in the BEV. The same threshold for hotspot was taken as in the automatic FIF case. The same weights were given to the sub-beams to remove the hotspot. The procedures of making subfields and adjusting beam weights are almost the same as for the automatic FIF case.

#### Dose index evaluation

*V*_105%_, *V*_95%_, *D*_95%_, *D*_max_, and the homogeneity index (HI) of the PTV in the semiautomatic FIF plans were compared with those in the original and manual FIF plans. *V*_105%_ was used as an index to indicate whether the hotspot decreased using the FIF technique. *V*_95%_ and *D*_95%_ were used as indicators of dose coverage. *D*_max_ was used as an indicator of excess dose. The dose and volume were expressed as the relative dose to the prescribed dose and relative volume to the PTV, respectively. *D_V_* was the dose to a specified fractional volume *V* in the PTV. *V_D_* was the volume that received at least the dose *D* in the PTV. The HI is defined as follows:



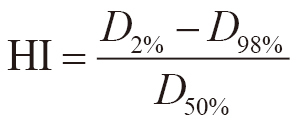
,


by normalizing the difference between *D*_2%_ and *D*_98%_ with *D*_50%_^16)^.

*V*_95%_, *D*_95%_, and *D*_max_ of the semiautomatic FIF and original plans and those of the semiautomatic FIF and manual FIF plans were statistically compared. Since these data did not follow a normal distribution, the Wilcoxon signed-rank test was performed using the JMP software (version 12.2.0; SAS Institute Inc., USA). A *P*-value of <0.05 was accepted as significant.

#### Measurement of the processing time for creating the sub-beams and adjusting the weights

The manual FIF creation time was measured with a stopwatch from the start of FIF creation to the end of the dose calculation. The elapsed time between the opening of the original plan in the TPS and the final dose calculation was measured in the automatic FIF script. The processing time included the time for creating sub-beams, adjusting the beam weights, and dose calculation.

## Results

### Comparison of dose indices and processing time

**Figure 6:**
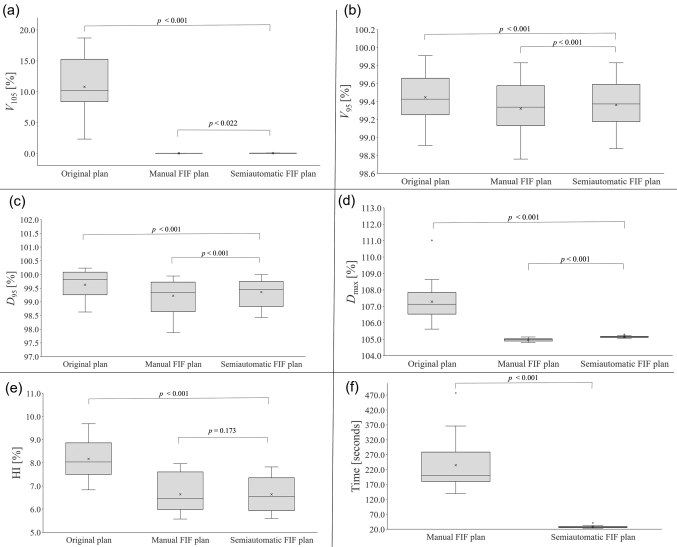
The box plots for (a) *V*_105%_, (b) *V*_95%_, (c) *D*_95%_, (d) *D*_max_, and (e) HI in the original, manual field-in-field (FIF), and semiautomatic FIF plans. The box plot for the processing time in the manual FIF and semiautomatic FIF plans is also presented in (f). p indicates P-values for the Wilcoxon signed-rank test.

A total of 21 cases resulted in FIF and one case in FIF-4SF. Figure 6a-e shows the box plots of *V*_105%_, *V*_95%_, *D*_95%_, *D*_max_, and HI in the original, manual FIF, and semiautomatic FIF plans. The creation times for the FIF plans are presented in Figure 6f. In manual FIF and semiautomatic FIF plans, *V*_105%_ decreased by almost 100% compared with the original plans, as shown in Figure 6a. This indicates that the hotspot region above *D*_th_ almost disappeared when using the semiautomatic FIF technique. *V*_95%_ values on average (± one standard deviation) for the original, manual FIF, and semiautomatic FIF plans were 99.4 ± 0.3%, 99.3 ± 0.3%, and 99.4 ± 0.3%, respectively (Figure 6b). Similarly, *D*_95%_ values on average for the original, manual FIF, and semiautomatic FIF plans were 99.6 ± 0.5%, 99.2 ± 0.6%, and 99.4 ± 0.5%, respectively (Figure 6c). *D*_max_ values on average for the original, manual FIF, and semiautomatic FIF plans were 107.3 ± 1.1%, 105.0 ± 0.1%, and 105.1 ± 0.1%, respectively (Figure 6d). The HIs values on average for the original, manual FIF, and semiautomatic FIF plans were 8.2 ± 0.8%, 6.6 ± 0.8%, and 6.6 ± 0.7%, respectively (Figure 6e).

The paired Wilcoxon signed-rank test indicated that there were significant differences in *V*_105%_, *V*_95%_, *D*_95%_, and *D*_max_ in the original and manual FIF plans compared with those in the semiautomatic FIF plans. There was also significant difference in HI in the original plans compared with that in the semiautomatic FIF plans.

The semiautomatic FIF planning times, which were defined as the processing times by the automatic FIF script to generate the sub-beams and dose calculations, were 25-29 s for FIF (Step 1) and 41 s for FIF-4SF (Step 2). The average time including FIF and FIF-4SF was 28 ± 4 s. The processing time for the semiautomatic FIF plan technique significantly decreased by 207 ± 84 s compared with that for the manual FIF plans (Figure 6f).

### Comparison of dose distribution

**Figure 7:**
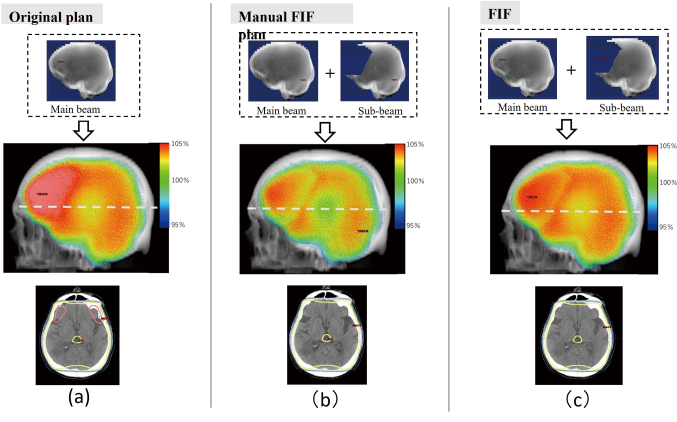
Dose distributions of the original, manual field-in-field (FIF), and semiautomatic FIF plans for case no. 22. (a) Field shape and dose distributions of the original plan. The upper, middle, and lower panels show the field shape, 3D dose distribution in the beam's eye view, and 2D dose distribution in a slice, respectively. The slice position is shown in the middle panel with a white dotted line. (b) Same as (a) but for the manual FIF plan. (c) Same as (a) but for the FIF plan in Step 1 (FIF). In the 2D dose distributions, the yellow lines are the 100% isodose lines, and the pink lines are the 105% isodose lines. The doses are relative to the prescribed dose.

**Figure 8:**
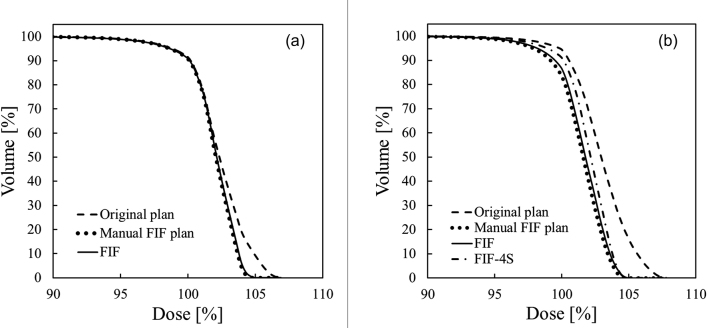
Dose volume histograms (DVHs) of planning target volume (PTV) in the original, manual field-in-field (FIF), and semiautomatic FIF plans. (a) DVHs of PTV in the original (dashed line), manual FIF (dotted line), and two-sub-beam FIF plans in Step 1 (solid line). (b) DVHs of the PTV in the original (dashed line), manual FIF (dotted line), FIF (solid line), and four-sub-beam FIF plans in Step 2 (FIF-4SF, dashed-dotted line).

Figure 7 shows a typical example of the dose distributions in the (a) original, (b) manual FIF, and (c) semiautomatic FIF plans for the case in Step 1. As shown by the dose distributions in the axial plane in Figure 7a and 6c, the 100% isodose line did not change in Step 1 even after the hotspot region exceeding 105% of the prescription dose disappeared using the semiautomatic FIF technique. The dose distributions for the manual and FIF plans were almost the same, as indicated in Figure 7b and c. The dose volume histograms (DVHs) of the PTV of the original, manual FIF, and FIF plans are shown in Figure 8a. *D*_max_ remarkably decreased in the manual FIF and FIF plans, while there was almost no change in *D*_95%_.

**Figure 9:**
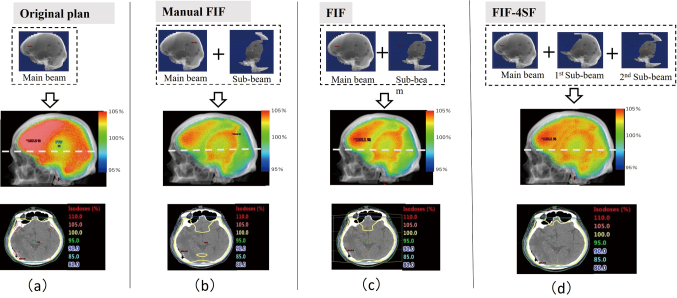
Dose distributions of the original, manual field-in-field (FIF), and semiautomatic FIF plans with the different numbers of sub-beams (FIF and FIF-4SF). (a) Field shape and dose distributions of the original plan. The upper, middle, and lower panels show the field shape, 3D dose distribution in the beam’s eye view, and 2D dose distribution in a slice, respectively. The slice position is indicated in the middle panel with a white dashed line. (b) Same as (a) but it is for the manual FIF plan. (c) Same as (a) but it is for the two-sub-beam FIF plan in the Step 1 (FIF). (d) Same as (a) but it is for the four-sub-beam FIF plan in the Step 2(FIF-4SF). In the 2D dose distributions, the yellow lines are the 100% isodose lines, and the pink lines are the 105% isodose lines. The doses are relative to the prescribed dose.

One case was categorized into Step 2 in this study. Figure 9 shows the dose distributions in the (a) original, (b) manual FIF, (c) FIF, and (d) FIF-4SF plans for this case. The FIF plan was rejected because the reduction in *D*_95%_ was >1% (1.6%), and Step 2 was performed. Figure 9d shows the dose distribution in the FIF-4SF plan. In the dose distributions of the manual FIF and FIF plans (Figure 9b and c), a low-dose region appeared in the field edge of the sub-beam. As shown in the axial image in Figure 9c, the 100% dose region in the FIF plan was smaller than that in the original plan. In the dose distribution in FIF-4SF (Figure 9d), the dose reduction around the field edge of the sub-beam was small compared with the dose distribution in the FIF plan.

Comparing the DVHs of the manual FIF, FIF, and FIF-4SF plans in Figure 8b, the maximum dose region that corresponds to the tail part of the DVHs is nearly similar in the three FIF plans. By increasing the number of sub-beams from two to four in the FIF-4SF plan, *D*_95%_ became close to the corresponding values in the original plan. The reduction of *D*_95%_ from the original plan was 1.6% in the FIF plan, and 0.7% in the FIF-4SF plan.

## Discussion

In this study, sub-beam creation and weight adjustment in FIF treatment planning were automated. These are the time-consuming parts in the FIF treatment planning and require ≥6 min if performed manually, as shown in Figure 6. The automatic script can be started with one click and requires <2 min, and reduces the burden on the treatment planner. Comparing the semiautomatic FIF and manual FIF plans, there was no significant difference in the HI; there were significant differences in *V*_95%_ and *D*_95%_. However, the differences of *V*_95%_ and *D*_95%_ were small. Thus, we found that the semiautomatic planning script could create the FIF plans almost equivalent to the manual FIF plans.

If a TPS has a scriptable interface, the proposed method can be easily introduced into the TPS. In this study, we adopted the change in *D*_95%_ as the criterion for selecting the FIF technique. The criterion can be easily modified by adjusting the parameters in the automatic FIF planning script, depending on the treatment site and protocol of each institution. The threshold value for the hotspot regions and number of sub-beams can also be changed. For example, if *D*_th_ is lowered, both *D*_max_ and *D*_95%_ will decrease. If the reduction of *D*_95%_ is acceptable, lowered *D*_th_ can be used. In this study, *D*_th_ was taken as 105% of the prescribed dose to make the maximum dose less than 107% of the prescribed dose considering the ICRU recommendation. *D*_th_ can be changed by considering a balance between *D*_max_ and *D*_95%_. In this study, the beam energies of the sub-beams were the same as those of the main beams (10 MV). The use of different energies for the sub-beams can be easily implemented and may improve the dose distribution.

Semiautomatic FIF planning was performed for the WBI cases, which were previously treated with the two-lateral-opposing-field technique. In the treatment plans with two-lateral-opposing technique, hotspot regions appeared in the frontal and occipital lobes, which are laterally thin parts of the brain^2, 4)^. Although there were significant differences in *V*_95%_ and *D*_95%_ between the original and semiautomatic FIF treatment plans, the average decreases in *V*_95%_ and *D*_95%_ were 0.1% and 0.4%, respectively. The effect of the reductions in *V*_95%_ and *D*_95%_ is expected to cause minimal deterioration of the quality of the semiautomatic FIF plans.

In this study, we adopted a 2-Step schema to select FIF technique. In the two-step FIF scheme, the number of sub-beams was increased from two to four to improve dose coverage of PTV, when the reduction in *D*_95%_ was >1%. One case resulted in the FIF-4SF plan. The reduction of *D*_95%_ compared with those in the original plan were 1.6% in the FIF plan. In the FIF-4SF plan, the reduction became 0.7% by increasing sub-beams. This indicates that the two-step FIF scheme can individualize the complexity of a treatment plan (beam number) depending on a patient.

The monitor unit (MU) for sub-beams becomes small by increasing the number of sub-beams. In the FIF plans, the weight of the main beam and sub-beam on the average was 0.979 ± 0.010 and 0.021 ± 0.010, respectively. In the manual FIF plans, the weight of the main beam and sub-beam on the average was 0.975 ± 0.011 and 0.036 ± 0.046. In the FIF-4SF plan, the weights of the main beams, first sub-beams, and second sub-beams were 0.973, 0.013, and 0.014, respectively. Small MU may cause dose uncertainty. To avoid the dose uncertainty due to small MU, the algorithm to limit the minimum MU is easily implemented in the automatic FIF script.

The average processing times were 27 s and 41 s for the FIF and FIF-4SF plans, respectively.

A longer time was required to create the FIF-4SF plan than the FIF plan. The average time of the 3 D dose calculation for the FIF plans was 19 s and the calculation time of the 3D dose calculation for the FIF-4SF plan was 28 s. The difference in the processing time was mainly explained by the time for the 3D dose calculation.

We used WBI cases to demonstrate the feasibility of the proposed method. Because the proposed method only requires a conventional plan without sub-beams as the input, it can be easily applied to other treatment sites, such as the whole breast^12)^ and esophagus.

Kim et al. reported on FIF planning automation for whole-breast irradiation^12)^. They used the ESAPI in their automatic technique. In their method, information of the original plan was exported to an executable program outside the TPS in the DICOM and DICOM-RT formats. In their method, the MLC shapes and beam weights for the sub-beams were calculated and automatically imported into the TPS. Using their method, FIF plans with the same quality as those manually created were obtained. We used almost the same approach as that of Kim et al., but our procedures can be performed within the TPS. Furthermore, we implemented several steps to personalize the treatment plan to the patient.

Yu et al. implemented an automated MLC shaping technique for WBI using deep learning. They could produce dose distribution almost equivalent to manually produced treatment plans. They used the two-opposing-lateral-field technique and obtained relatively high maximum dose of approximately 110%. By combining their technique with ours, a fully automated treatment plan for WBI can be realized.

By automating the sub-beam shape and adjusting the weight, these processes can be standardized^17)^. The manual shaping of sub-beams and adjustment of beam weights depend on the skill and preference of the treatment planners. The automation of FIF planning can reduce the variability originated from the skill and preference of the treatment planners and the possibility of human error. However, because the transition from manual to automated planning can potentially lead to systematic errors that are difficult to detect^18)^, the final verification must be performed by humans.

A limitation of the present study is all manual FIF plans were created by one physicist. The processing time and the quality of the FIF plans probably depend on the experience of treatment planners. In this study, the similar plans were obtained for the manual and automatic FIF planning. One reason of this similarity is probably that a single person made the manual FIF plans. The manual plans would diverge from the automatic FIF plans if multiple persons created the manual plans. However, the indication that the automatic FIF creation reduces the burden of the treatment planners is still valid because the sub-beam creation and weight adjustment are automatically performed.

We developed a semiautomatic FIF planning method and implemented it in a TPS. By applying it to WBI, we confirmed that the semiautomatic FIF technique could reduce hotspot regions with a slight change in the PTV coverage compared with the original plan. When combined with a selection of an FIF scheme individualized to each patient, its performance was equal to or better than the manual FIF plan.

## Funding

This study was funded by JSPS KAKENHI (grant number JP15K08702).

## Author contributions

HW, SS, TK, HN, TI, CK, KU, JT and KS participated in the study design and data interpretation. Satoru Sugimoto performed manual treatment planning. KK participated in treatment planning data collection. HW wrote the draft manuscript. SS, TI, JT and KS reviewed and revised the draft and final manuscript. All authors read and approved the final manuscript.

## Conflicts of interest statement

There are no conflicts of interest in relation to this study.
